# Strengthening research-extension-farmer-input linkage system for sustainable smallholder livestock farming in Africa: progress and prospects

**DOI:** 10.1007/s11250-024-04210-9

**Published:** 2024-10-28

**Authors:** Obvious Mapiye, Kennedy Dzama

**Affiliations:** Department of Animal Sciences, Faculty of AgriSciences, Stellenbosch University, Stellenbosch, South Africa

**Keywords:** REFILS, Agricultural research, Linkage, Agricultural extension, Smallholder livestock farming

## Abstract

The drive to hasten the development of smallholder livestock farming through research-extension-farmer-input linkage systems (REFILS) is being promoted in African countries. Resource-constrained smallholders face various challenges including poor access to timely and relevant research-based innovations. Weak research-extension-farmer-input linkages exacerbate this issue. This review explores the evolution of Knowledge Transfer and Agricultural Extension Theories, emphasising the shift from centralised top-down dissemination to participatory and user-driven models. The paper characterises “research”, “extension”, “farmer”, and “input” as the main pillars of agricultural knowledge systems and how their lack of interconnectedness impacts their effectiveness. Examining the dynamics of these pillars provides a comprehensive rationale for strengthening REFILS. Also, REFILS adoption disparities such as limited funding and investment, institutional and organisational inefficiencies, and social and cultural factors were discussed. The identified key challenges form an intricate web of interconnected issues which should not be addressed in isolation but collectively. The proposed measures for REFILS enhancement include building strong public-private partnerships, full participation and collaboration by all key actors including farmers, digitalisation of smallholder agriculture, and policy and institutional reorganisation towards a stronger REFILS. Moreover, the study acts as a foundational guide for strengthening REFILS at national, regional, and continental levels to foster economic, environmental, and social sustainability in Africa’s livestock industry.

## Introduction

Strengthening the synergy between research, extension, farmers, and input linkage systems (REFILS) stands as a pivotal strategy to unlock the potential for agricultural innovation and achieve sustainability within smallholder farming (Nnadozie et al. 2015; Belay and Dawit 2017; Food and Agriculture Organisation (FAO) 2020). According to Landini et al. (2023) and Modirwa and Oladele (2017), the promotion of innovation and development within agricultural systems depends on the strengths of linkages among the main stakeholders involved. The relationship between research as innovators, extension services as transmitters of technologies, livestock keepers as consumers of these technologies, and input suppliers as manufacturers of production inputs, commonly referred to as REFILS has long been studied and analysed. Also, the advantages of strong REFILS are well-known as acknowledged by van Crowder and Anderson (1997). It appears, however, that this complex relationship between REFILS actors remains disconnected despite efforts to make it work (Nyamupangedengu and Terblanché 2016; Sigman 2016; Saravanan and Suchiradipta 2017). This disconnection further heightens the rate at which agricultural research innovations are lost before they are accessed by farmers (Nnadozie et al. 2015). Nonetheless, it is important also to note that collaboration and coordination are not easy in practice, especially in the agriculture sector, where stakeholders are many and roles are fluid (Saravanan and Suchiradipta 2017). This calls for strategies that effectively synergise agricultural research and knowledge transfer processes to align with the needs of farmers and other ecosystem players.

Agricultural knowledge development and dissemination have played a pivotal role in shaping new policies and strategies for rural livestock development. This review delves into the transformation of Knowledge Transfer and Agricultural Extension theories, highlighting their historical progression and key contributions. This demonstrates their dynamic evolution from top-down and supply-driven approaches (Alex et al. 2002; Swanson and Rajalahti 2010; FAO 2017) to participatory and collaborative approaches (Cai and Davis 2017; MacNairn 2018; Agwu et al. 2023). These theories have adapted to the evolving agricultural landscape and its challenges and are progressively recognising the need to synergise all actors from technology creation to end-users (Nyamupangedengu and Terblanché 2016).

Smallholder livestock farmers predominantly participate in REFILS programmes as passive recipients, and this has adversely affected their performance. Smallholder livestock farming is not merely an economic activity in Africa; it also encompasses cultural and social aspects, supplies of dietary protein, and the overall protection of vulnerable households (Shava and Masuku 2019). Despite this, smallholder livestock farming in Africa faces several critical challenges, notably limited access to relevant and timely research-based knowledge by farmers. Baig and Aldosari (2013) argue that most African smallholder livestock producers reside in remote and resource-constrained regions resulting in poor access to agricultural knowledge and technology. Therefore, such farmers continue to be unaware of new research findings and innovations that could significantly increase their productivity and sustainability (Sigman 2016). The continued inadequate transfer of research findings to farmers consequently limits their ability to address everyday challenges and respond positively to opportunities for growth and sustainability (Khwidzhili and Worth 2019). This could be explained by the absence of effective linkages between local agricultural researchers, extension officers, smallholder farmers, and other stakeholders (Nyamupangedengu and Terblanché 2016; Saravanan and Suchiradipta 2017).

Considering this challenge and recognising the vital role of effective REFILS, this paper explores these synergies in the context of African smallholder livestock farming. Using a critical analysis of this nexus, the review examines how research and technology development, dissemination, and adoption by farmers are aligned. For farmers to access the best technologies and practices, whilst bridging the knowledge gap, all stakeholders must work together (Sigman 2016). Furthermore, investigating the systemic challenges weakening REFILS and obstructing sustainable agricultural innovation systems (AIS) is at the centre of this study (Landini et al. 2023; Modirwa and Oladele 2017). Ultimately the study provides a robust rationale for examining the dynamics of REFILS, thereby bolstering the systemic capacity of national, regional, and continental livestock sector stakeholders.

### Knowledge transfer in African livestock agriculture

Knowledge transfer theory is a multidisciplinary framework that plays a pivotal role in understanding how knowledge is disseminated and applied across various domains, including livestock agriculture. Rooted in the broader field of knowledge management, this theory seeks to explain the mechanisms and factors that influence the movement of knowledge from one source to another, as well as its subsequent utilisation (Cong et al. 2007; Thomas and Pretat 2009). Argote et al. (2000) view the transfer of learning material as a dynamic process and define knowledge as a product of the process. According to Seidman and McCauley (2005), knowledge involves the cumulative experiences, attitudes, and skills developed to enable individuals to perform a function consistently, systematically, and effectively. This is what makes “knowledge” a unique good that varies from individual to individual (Thomas and Pretat 2009). In agriculture, knowledge is one of the four factors of production after labour, land, and capital as it determines farmer management ability (UNDP 2012).

Knowledge transfer theory postulates that knowledge is not static but a dynamic entity that can be exchanged, modified, and adapted as it moves from one context to another (Szulanski 1996). This theory draws from diverse disciplines emphasising the importance of both tacit and explicit knowledge in knowledge creation and transfer processes (Cong et al. 2007). Tacit knowledge is highly personal and subjective, experience-based, and challenging to articulate, while explicit knowledge is codified and can be easily communicated (Nonaka and Takeuchi 1995). Therefore, knowledge management drives the integration of explicit and tacit knowledge among farmers. More importantly, it embraces all processes and practices associated with the creation, acquisition, sharing, and use of knowledge including skills and expertise (Cong et al. 2007). Therefore, effective knowledge management is increasingly considered a basis for sustainable competitive advantage (Thomas and Pretat 2009).

Historical and early approaches to knowledge transfer theory in African livestock farming reflect a complex interplay between traditional knowledge, colonial influences, and evolving approaches to disseminating agricultural knowledge (Swanson and Rajalahti 2010). Understanding this background sheds light on the challenges faced and the innovations employed in the transfer of knowledge to improve livestock agricultural practices and productivity. Many challenges have historically hindered effective knowledge transfer (Argote et al. 2000) and subsequently the technical efficiency of farmers (Tanko and Ismaila 2021). Some of the key challenges faced include cultural and religious disparities, limited access to resources, gender disparities and socio-economic factors (Kebebe 2019; Lubungu and Birner 2021). Szulanski (1996) identified the recipient’s insufficient absorptive capacity, causal ambiguity, and the challenging nature of the relationship between the source and recipient as significant obstacles to knowledge transfer. Tanko and Ismaila (2021), noted the imposition of external knowledge which often clashes with traditional practices and beliefs, leading to resistance and reluctance to adopt new approaches. Gender inequalities in access to and control over resources and decision-making further compound the challenges faced by female cattle farmers in accessing new knowledge (Lubungu and Birner 2021; FAO 2017). Reducing inequality and enhancing access to new knowledge by women can boost agricultural productivity and alleviate poverty in the region. This is because women are directly engaged in over 60% of agricultural activities within the smallholder systems (Tsan et al. 2019). Furthermore, Baig and Aldosari (2013) highlighted economic disparities and unequal access to education and extension services as challenges in the adoption of modern farming practices.

In hindsight, the discussed issues regarding effective knowledge transfer have necessitated adaptations to contemporary knowledge transfer approaches, promote stakeholder participation, recognition of indigenous knowledge, sustainable and climate-smart farming, and the integration of Information and Communication Technology (ICT) tools, such as mobile and internet-based platforms (World Bank 2017; Mapiye et al. 2021). Also, in navigating these complexities, this paper emphasises the importance of ongoing efforts to further develop the extension theory as a key bridge for the effective transfer of research-based knowledge to smallholder farmers.

### The agricultural extension concept and its evolution in Africa

There is no ostensive definition for exclusively defining the agricultural extension concept because the views of what it is have continuously evolved. Oakley and Garforth (1985) defined agricultural extension as the means of introducing new knowledge and ideas to farmers and farming communities for improved production and lifestyle standards. Alex et al. (2002) broadly defined it as… “the rural knowledge and innovation system.” Duvel (2004) broadened this definition to a collaborative process involving farmers, aimed at providing them with relevant skills and information to bolster productivity and sustainability.

Agricultural extension theory emerged in the late 19th century with initiatives like the Cooperative Extension Service in the United States. Historical accounts reveal that formal agricultural extension was introduced to Africa during the colonial period (Swanson and Rajalahti 2010). However, during the pre-colonial era, African farming knowledge was primarily transferred through oral traditions and communal practices through elders from generation to generation (Ikechukwu and Ejikemeuwa 2020). Mapiye et al. (2021) describe early extension theories as primarily concerned with technology transfer or extending research-driven knowledge to rural livestock farming areas. These early extension efforts followed a top-down and linear model, where experts and government agents dictated the knowledge transferred to farmers (Nagel 1997; Swanson and Rajalahti 2010; FAO 2020). Because these approaches were not participatory and did not acknowledge traditional indigenous knowledge and user preferences, their successes were limited.

There has been an evolution in African agricultural extension theory towards participatory and community-centred approaches over the years (Vaarst et al. 2007; GFRAS 2016). It has attempted to move beyond technology transfer to facilitation, from teaching to learning, and from individual farmers sharing information to collectively sharing information (Baig and Aldosari 2013; Agwu et al. 2023). The emerging concepts also include advisory services, rural development, and addressing socio-economic challenges (Swanson and Rajalahti 2010; Mapiye et al. 2021). Participatory-oriented and pluralistic extension strengthens farmers’ involvement and the need to create extension solutions based on their diversified and specific needs (Vaarst et al. 2007; Baig and Aldosari 2013; Nwafor et al. 2021). For these reasons, the definition of extension should acknowledge that there is a growing body of knowledge that advocates the recognition of livestock farmers’ knowledge and experience of their farming systems. It also advocates proactively incorporating it into extension systems. Instead of using the term extension agents, some scholars (Nwafor et al. 2021; Agwu et al. 2023) prefer using the phrase agricultural advisory services, because the latter is in a much broader context. The current review proposes the term ‘agricultural learning and advisory services’ in recognition of the broader context and the fact that ‘learning’ is a three-way phenomenon that should include livestock farmers and ‘advisors’ in an extension system and also researchers generating the technologies.

Furthermore, agricultural extension theory has broader responsibilities as it now emphasises agricultural sustainability and innovation (Sigman 2016; Khwidzhili and Worth 2019). Sustainability is central to addressing environmental concerns, ensuring long-term agricultural viability (profitability), and reflecting on food quality and public welfare concerns (Saravanan and Suchiradipta 2017). However, incorporating sustainability principles requires better skills by researchers, extension officers, farmers, and other stakeholders because sustainable agriculture is increasingly becoming a knowledge-intensive concept (FAO 2017). Also, contemporary extension theory includes livestock innovation systems and market-oriented approaches (Chander and Rathod 2015; Sigman 2016; Nwafor et al. 2021). A framework for the agricultural knowledge and innovation system (AKIS) helps to stimulate innovation and enhance synergies and coordination among all actors (Zahran et al. 2020). This improves extension theory by facilitating knowledge exchange and accelerating progress through an agricultural innovation system (Saravanan and Suchiradipta 2017). Therefore, embracing AKIS within extension ensures farming remains competitive, adaptive, and aligned with new challenges and opportunities for growth. A growing recognition of the importance of contextualised solutions for livestock farming drives these shifts in African agricultural extension theory (Mapiye et al. 2021). Moreover, the integration of ICT has played a pivotal role in modernising African agricultural extension (Marwa et al. 2020; Mapiye et al. 2021). Mobile phones and the internet are revolutionising the dissemination of extension knowledge and making it more accessible to rural communities (Tsan et al. 2019).

The evolution of extension systems reflects a more informed understanding of the diverse and systemic challenges faced by smallholder livestock farmers and the need for responsive and sustainable extension approaches. In Africa, agricultural extension services are provided to smallholder livestock farmers by multiple players and through numerous approaches (Mapiye et al. 2021), and understanding the major approaches used is critical for their improvement.

### Agricultural extension approaches for supporting livestock production in Africa

In Africa, there are many multifaceted approaches to extension. Several authors have classified them differently and contributed to their theoretical understanding (Table 1). Due to the diversity of farmer needs, production systems, and agricultural policy objectives, all countries employ a mix of extension approaches (Nwafor et al. 2021). Thus, over the past few decades, extension has been tested and deployed using different approaches and strategies (Marwa et al. 2020). These systems have evolved, as they attempted to adapt to the unique challenges and opportunities facing the continent’s diverse agricultural landscape. Invariably the approaches are not mutually exclusive and can be complementary (Mapiye et al. 2021). Table 1 identifies the various classifications of extension approaches used to support smallholder livestock farmers in Africa. This also includes, their type, focus, tools, and institutional arrangements. The approaches broadly include technology transfer, commodity specialisation, participatory extension, cost-sharing, and education institutions (Mapiye et al. 2021).

Extension approaches vary from pure classical or conventional extension with a top-down approach [Training and Visit (T&V)] to those including significant farmer participation [Farmer Field Schools (FFS)] and those that integrate agricultural research, extension, and farmer participation [Farming Systems Research and Extension (FSRE)]. One prominent approach is the T&V system, introduced with sponsorship from the World Bank in Africa (Swanson and Rajalahti 2010). This approach emphasises regular visits by extension agents to farmers, providing technical advice and facilitating technology transfer. Additionally, the concept of FFS was championed in Asia to promote Integrated Pest Management and gained recognition in Africa. Farmer Field Schools emphasise experiential learning and farmer-to-farmer knowledge sharing (Vaarst et al. 2007). Despite having been introduced many years ago, some of these pluralistic extension systems engender the involvement of multiple stakeholders, including government agencies, Non-Governmental Organisations (NGOs), and the private sector, to diversify extension services and reach a broader range of farmers (Swanson and Rajalahti 2010; Nwafor., et al., 2021). Another significant dimension to consider is the role of indigenous knowledge systems. Mugwisi (2017) highlighted the importance of incorporating traditional practices and local wisdom into extension strategies to make them more contextually relevant.

The discussed extension theory provides a foundation for understanding the dynamics of knowledge transfer, which is integral to collaborative efforts needed in agricultural research and technology development. This synergy is crucial for addressing the knowledge gap, enhancing technology adoption, and ultimately bolstering the resilience and productivity of smallholder livestock farming systems in Africa.

**Table 1:** Agricultural extension approaches, types, focus areas, tools and institutional arrangements

Extension approach	Type	Focus	Tools	Institutional arrangements
Training and Visit approach (T&V)	Ministry-based	Technology transfer; livestock husbandry practices	Training modules; farmer visits; field-days; field demos	Ministry of Agriculture; government agencies; other players
Public extension	Ministry-based	Technology transfer; livestock agricultural policies	National policy tools; farm visits; group training	Ministry of Agriculture; central government; other players
Commodity-based extension	Commodity-based	Promote high-income livestock projects; predominantly export products; strategic agricultural inputs	Planned extension modules; coordination; farmer contracts; livestock commodity	Centralized government coordination; private organizations; contracted farmers; research universities; parastatals
Farmer Field School Approach (FFS)	Participatory	Livestock agriculture; Integrated Pest Management	Group learning; experiential learning; observations	NGOs; government agencies; agricultural research institutions
Project (Integrated) Extension	Project-based	Poverty alleviation; technology demonstration	Training manuals/guides; field days; demonstration farms	Central government; international development agencies; local extension system
Farming Systems Research-Extension	Participatory	Agriculture; livestock farmers’ needs; technology development and testing	On-farm research projects; lead farmers; research institutions	Government department of research and extension; private sector
Cost-sharing extension	Formal, cost-oriented	Livestock farming	Livestock projects; user fees; government subsidies	Private companies; central, regional and local government units; individual farmers
Education institution extension	Formal decentralized	Farmer empowerment; practical knowledge transfer; technical skills development	Classroom teaching; field demos; curriculum development; policy briefs; research outputs	Educational institutions (Agricultural colleges, universities); farmer groups
Private extension	Formal- private	Specialized livestock technical expertise; business skills; market access	consultancy sessions; workshops; product demonstrations; websites	Private companies; consulting firms; agribusiness associations; private and public training institutions

*Sources* (Rivera 1988; Axinn 1988; Nagel 1997; Eicher 2007; Abdu-Raheem and Worth 2016; Mapiye et al. 2021)

### Agricultural research and technology development

Agricultural research stands as one of the earliest forms of organised research globally. Livestock research spans various levels, from molecular studies to practical applications and health maintenance (Townsend and Thirtle 2001). In many countries, National Agricultural Research Systems (NARS) exist which encompass livestock researchers at universities, public institutions, the private sector, and related institutions (Bingen and Gibbon 2012). However, the performance of these institutions remains sub-optimal in many parts of the world due to various factors like inadequate funding and misaligned research priorities with farmers’ needs (FAO 2020).

In recent years, agricultural research efforts have also focused on sustainability (FAO 2020; Khwidzhili and Worth 2019). The research mission has significantly moved beyond merely enhancing yields, but also considering the long-term environmental impact of farming practices and accounting for the socio-economic dimensions of sustainability, including technological, spatial elements, and the empowerment of marginalised groups (Loebenstein and Thottappilly 2007; Saravanan and Suchiradipta 2017). Also, livestock research aims to help foster innovations and develop better policies which are key drivers for advancing agricultural productivity and building resilience within the sector (Chander and Rathod 2015; FAO 2020). For instance, research-backed livestock production innovations have helped to increase productivity and improve resilience against pests and diseases (Quddus 2022). This was largely in the commercial farming sector and key questions continue to be raised about how these advances have been transferred to smallholders who are central to African livestock agriculture.

While on paper, the process of conducting research, packaging it to fit local contexts or farmers’ needs, and delivering it through extension seems straightforward, it is more complex in practice (Sigman 2016). The key question is that of the location, where should livestock research be carried out? In recent years there have been efforts to conduct research on-farm and introduce an element of co-creation/ farmer participation and hence buy-in from all stakeholders. For instance, FSRE proponents (Bingen and Gibbon 2012) have always argued in support of on-farm research for these reasons. As prudent as this argument may seem, some research cannot be done on farms, and will still need to be conducted at research stations and laboratories. This applies to much of the basic research which is important and sometimes not highly visible but underpins most of the applied research.

Another big question has been about agenda setting. Who decides what research should be done? Quite often, there is a misalignment between the research being conducted and the challenges farmers face. This results in farmers being forced to consume or adopt research and technologies that may not be appropriate for them. This top-down approach has characterised agricultural research and often resulted in failure (Loebenstein and Thottappilly 2007; Agwu et al. 2023). For example, big livestock breeding companies have often come up with genotypes that do not match farmers’ needs and enormous amounts of money had to be spent on behaviour-changing efforts to sell such products. Agricultural innovations respond better to local challenges when they are co-created through participatory processes (GFRAS 2016). In support of these arguments, Mugwisi (2017) noted that the new research paradigm needs to consider and incorporate local indigenous technical knowledge (ITK) systems and biodiversity rather than focusing on external phenomena. The challenge has always been to strike a balance between modern scientific research and ITK (Swanson and Rajalahti 2010). In addition, extension agents should also be involved in identifying and prioritising research goals based on their knowledge and understanding of farmers’ challenges (Baig and Aldosari 2013; Kebebe 2019). This will increase the usability and adoption of livestock research-driven technologies. The location of the research may not matter if these conditions are met. Lastly, market-oriented agriculture research is necessary due to the changing dynamics of local, regional, and global market trends.

A key point of this discussion, livestock research is viewed as an integral component of the agricultural system. However, the tendency has been to insulate and elevate research above the other components leaving it largely esoteric. This has led to the formation of a hierarchy or pyramid where research is above extension with farmers at the bottom. This is the major reason why many livestock research and development systems remain entrenched in silos and top-down operations. To improve livestock research and technology creation, the synergy between farmers, extension agents and researchers, as well as other key stakeholders in the agricultural value chain needs to be strengthened.

### African smallholder livestock farmers and technology uptake

In Africa, livestock farms are diverse in scale and can range from small-family subsistence farms to large-scale outfits run on intensive, semi-intensive, and extensive industrial scales. According to FAO (2017), the main types of livestock commonly raised on African smallholder farms are cattle, goats, sheep, poultry, pigs, and camels, depending on the region’s ecological and cultural factors.

Livestock farmers receive research-developed technologies directly from researchers or via extension agents. Moreover, farmers also contribute to both research and extension by extending technologies among themselves and by developing new or adapting existing ones. A study by Mapiye et al. (2019) showed that livestock farmers were reported as the second most important source of information after public extension. Farmers should engage in participatory research and collaborate with scientists and extension personnel to adapt innovations to their local farm conditions (Sigman 2016; Cai and Davis 2017). Farmers benefit from this approach as it promotes the co-creation of knowledge and aligns innovations with their needs and practices (Bellon et al. 2011).

Multiple interlinked factors influence African smallholder livestock farmers’ uptake of research and extension innovations. Accessibility challenges in remote areas, coupled with inadequate infrastructure and limited private sector involvement in inputs and markets, impede innovation adoption (Nwafor et al. 2021). Constraints within public extension services, including resource limitations and outreach constraints, contribute to reduced awareness of research-based innovations among livestock farmers (Sigman 2016). Additionally, low skills and limited education hinder farmers’ comprehension and access to extension services, creating barriers to adopting sustainable practices. Cultural beliefs deeply entrenched in local traditions can further impede technology adoption, as traditional knowledge and religious beliefs may discourage change even when research-backed innovations offer benefits (Tanko and Ismaila 2021). Future efforts should focus on building robust partnerships between farmers, scientists, and extension personnel, facilitating the seamless integration of research-driven solutions into the agricultural landscape. This approach aims to address the integrated challenges encountered by farmers, promoting sustainable innovation uptake.

### Research-extension-farmer-inputs linkage system (REFILS)

Research, extension, farmers, input suppliers and other actors form the key pillars of agricultural knowledge systems, and their impact largely depends on strong synergy among each other. The REFILS theory emerged in response to the need for a holistic approach to agricultural development (Swanson and Rajalaht 2010) and has since shaped agricultural policies and practices globally and in Africa. It underscores the importance of effective communication and working relationships, feedback mechanisms, and adaptive approaches established between two or more individuals and/or organisations in the system (Agbamu 2000). This theory has evolved, recognising the need for context-specific solutions that address the multifaceted challenges faced by livestock farmers in different regions and farming systems. On paper, this framework is quite logical and supposed to work well, but in practice, this is not often the case. In fact, van Crowder and Anderson (1997) and Sigman (2016) noted that technology transfer from researchers to farmers through extension is a persistent and pervasive problem.

In Africa, besides government funding, there are government-led extension services supported by private funding that disseminate research findings, offer technical advice, and deliver inputs to farmers (Nwafor et al. 2021). REFILS models involving the private sector are increasingly being advocated for as they leverage private sector resources, expertise, and market access to deliver research-based innovations to farmers (Nwafor et al. 2021). The Alliance for a Green Revolution in Africa (AGRA) engages both public and private partners to enhance agricultural productivity (AGRA 2021). There are participatory approaches that emphasise farmer involvement in REFILS processes (GFRAS 2016). Old methods like FFS and Participatory Rural Appraisal (PRA) engage farmers in knowledge co-creation and problem-solving. In Africa, FFS programs have been implemented in various countries, such as Kenya, Tanzania, and Uganda (Vaarst et al. 2007).

According to Agbamu (2000), there are five types of REFILS, as follows:

1. Research and extension organisations operate at the same status in a country, using a bottom-up approach in decision-making on linkage activities.
2. Both organisations have the same operative status, using a top-down approach to managing the links.
3. Research and extension organisations have unequal status, and the linkage system operates according to a bottom-up management approach.
4. Both organisations are unequal in status, and the linkages operate according to a top-down management approach.
5. There is no organised linkage system between agricultural research and extension organisations.

Moving forward, strengthening the REFILS requires a concerted effort to foster and enhance two-way communication channels, and foster collaboration among diverse stakeholders. Also, the decentralisation of research and extension services is a prerequisite of these models especially where the bottom-up approach of involving farmers in identifying problems for research and decision-making is in place. Before any measures can be implemented, it is important to understand some of the key challenges hindering and weakening the synergy among the systems’ players.

### The extent of REFILS adoption and its implementation constraints in livestock agriculture

The state of REFILS in Africa is characterised by both challenges and promising developments and its adoption remains mostly poor across the continent. Table 2 presents the state of REFILS in various selected African countries. Adoption rates vary across African countries due to differences in infrastructure, funding, and policy support levels. For instance, countries like Kenya, Uganda, Rwanda, and Ethiopia have shown substantial progress in integrating research, extension services, and farmers (Belay and Dawit 2017; MacNairn 2018). Despite that, weak linkages have been reported in various countries of Africa (Landini et al. 2023; Agwu et al. 2023) including big economies like Nigeria (Olatunji et al. 2015) and South Africa (Raidimi et al., 2017).

**Table 2:** State of REFILS in selected African countries

Country	State of REFILS	Reference
Ghana	State of REFILS is characterised by poor funding, and poor policy environment.	Doamekpor (2006)
Egypt	Disconnection among the key REFILS components. There is a strong call to build innovation into the system.	Zahran et al. (2020)
Nigeria	REFILS was introduced by donors. Participation declined once donor funds dried up. Attempts to resuscitate the program underway but poorly funded.	Olatunji et al. (2015)
Zimbabwe	Historically good links between REFILS role players. Currently, there are very weak linkages between farmer-extension services, farmer-researchers and researcher-extension services.	Nyamupangedengu and Terblanché (2016)
Tanzania	Unequal status between extension and research and poor funding characterises REFILS. Decentralisation of system can help linkage between research and extension.	Agbamu (2000)
Tunisia	Public platform exists to promote REFILS, but participation is limited to government only. There is little or no role for NGOs, farmers and private sector.	Melaouhia et al. (2015)
Ethiopia	Strong and explicit structures in place to promote REFILS. Moving towards equal status between research and extension. Challenges still exist in the form of inadequate funding and poor farmer participation.	Belay and Dawit (2017)
Rwanda	Strong linkages among REFILS role players, with merged research and extension services, high farmer participation, and substantial donor support.	MacNairn (2018)
South Africa	Weak extension but strong research. Poor linkage between the two and farmers. Key policy documents not strong on REFILS. Strong emphasis on sustainable agriculture	Mapiye et al. (2019); Khwidzhili and Worth (2019)
Uganda	Disconnection among the key components and characterised by poor funding. Technologies being extended not matching farmer needs. Gender insensitive. Strong bias towards crop agriculture.	Davis et al. (2020)
Malawi	Poor funding, inappropriate and outdated technologies being extended as a direct result of lack of linkages between key components.	Cai and Davis (2017); Agwu et al. (2023)

It is concerning that research, extension, farmers and other key stakeholders continue to function with very little collaboration (Sigman 2016; Belay and Dawit 2017). Modirwa and Oladele (2017) in their study on the impact of linkage activities on the collaboration of REFILS stakeholders, extension officers exhibited a higher level of engagement in such activities compared to other stakeholders. This indicates a discernible gap in the involvement of different stakeholders in linkage activities. Also, Zwane (2020) on AIS in Limpopo found that extension officers face challenges in meeting farmers’ diverse needs extending beyond information on inputs and markets. Thus, the historic gap between REFILS actors continues to hamper efforts aimed at disseminating improved technologies and practices to livestock farmers (Sigman 2016). The challenge persists even though some countries, such as Zimbabwe and Gambia, have merged research and extension departments (Nyamupangedengu and Terblanché 2016). While efforts are being made to strengthen REFILS in African livestock agriculture, various systemic challenges (Table 2) continue to constrain progress, and these should be identified and eliminated.

#### Inadequate funding and support resources

One of the primary factors driving the weakening and slow adoption of REFILS is the chronic underfunding of agricultural research and extension services by many African governments (Belay and Dawit 2017; FAO 2020). Insufficient financial support limits research institutions and extension services’ capacity to work together in conducting meaningful research, developing innovative technologies, and reaching out to farmers effectively (Alex et al. 2002; Technical Centre for Agricultural and Rural Cooperation (CTA) 2011; FAO 2020). Nigeria previously had a well-structured REFILS programme funded by the World Bank’s National Agricultural Research Project programme from 1995 to 2000 (CTA 2011; Olatunji et al. 2015) (Table 2). However, these public extension systems started to weaken due to financial shortages after the withdrawal of funding by the World Bank (Alex et al. 2002).

In Uganda, Ethiopia, and the Democratic Republic of the Congo, Davis et al. (2020) found that extension services faced insufficient funding and frequently depended on donor support which lacked sustainability. As shown in Table 2, the functioning of REFILS in countries like Ghana have been affected by poof funding since two decades ago (Doamekpor 2006). Insufficient funds hinder the performance of REFILS stakeholders, particularly in terms of operational expenses and staffing levels. Also, the transition of Malawi’s agricultural policy of 2000, aiming to drive the involvement of the private sector, civil society, and farmer-based organisations in extension and advisory services (EAS), has been hindered by inadequate financial support (Cai and Davis 2017). Likewise, inadequate resource allocation including transport and a shortage of qualified and motivated personnel in both research and extension agencies impede REFILS and its adoption in various countries (Davis et al. 2019). In Nigeria, the extension-to-farm family ratio in 2019 varied from 1:5000 to 1:10000 (Davis et al. 2019). Ethiopia despite having one of the highest development agent/extension-to-farmer ratios in Africa has a shortage of skilled extension personnel which hinders effective knowledge dissemination to farmers (Brhane et al. 2017). The capacity limitations highlight the need for financial investment in bolstering private sector involvement, collaborative activities and training and recruitment programmes (Belay and Dawit 2017; Nwafor et al. 2021). This will strengthen REFILS linkages and activities across all levels.

#### Institutional and organisational ineffectiveness

Insufficient institutional and organisational structures in the agricultural sector create obstacles to REFILS implementation. Invariably, complex administrative setups, bureaucratic processes, and lack of coordination and accountability among public research institutions, and government extension agencies lead to fragmented efforts (Saravanan and Suchiradipta 2017; Agwu et al. 2023). For example, in Malawi, major actors providing EAS were found to operate independently without strategic and operational collaboration with other stakeholders and had limited interactive processes with the private sector (Cai and Davis 2017; Agwu et al. 2023). A study by Nyamupangedengu and Terblanché (2016), in the Nyanga district of Zimbabwe found non-existent farmer-research linkages as well as disengaged overall farmer-extension and research-extension linkage systems that lacked coordination. In Egypt the disconnection among stakeholders have been found to negatively impact REFILS implementation (Zahran et al. 2020) (Table 2).

Similarly, Belay and Dawit (2017) highlighted challenges related to the insufficiency of internal quality control systems in implementing REFILs in Ethiopia, despite positive progress. Inconsistent policies also deter private sector involvement in agricultural research and extension, limiting services available to farmers (Nwafor et al. 2021). For instance, many public research institutions continue to perform sub-optimally owing to factors such as lack of funding, poor management, lack of human capacities, weak collaboration with EAS, high dependency on donor funding and not being demand-driven (FAO 2020). To overcome these challenges, policy, and organisational reforms are crucial to ensure improved coordination and coherence by all players at the national, provincial, and local levels (Belay and Dawit 2017; Agwu et al. 2023).

#### Market and input access challenges

The extension systems in sub-Saharan African (SSA) often includes market-oriented extension and advisory services, but these services lack the necessary capacity and information to be successful (Davis et al. 2020; Nwafor et al. 2021). Limited access to markets and inputs significantly weakens the sustainability of REFILS, especially in the context of smallholder livestock farming. When farmers lack access to essential inputs such as high-quality adapted breeds, and veterinary drugs and services, their productivity and income potential are severely hampered. Similarly, limited access to markets prevents farmers from selling their products at competitive prices. This discourages them from investing in improved farming practices or adopting new technologies.

#### Social and cultural factors

Social and cultural factors can also contribute to the inability of farmers to benefit from REFILS initiatives. Traditional beliefs, practices, and norms within farming communities may resist change, making it challenging to introduce and promote new research technologies (Tanko and Ismaila 2021). Gender disparities can further exacerbate these challenges, as women may have limited access to extension services and resources (Lubungu and Birner 2021; Agwu et al. 2023). In a study by Lubungu and Birner (2021) on gender relations in smallholder cattle production in Zambia, quantitative findings indicate that while women may own cattle, they are less likely to expand their cattle ownership share due to anticipated intra-household conflicts. Also, the onset of transitioning from traditional to modern farming systems in Uganda has been a challenge (Matsumoto et al. 2013) owing to traditional influences which negatively affect the farmers’ willingness to adopt new technologies. Despite the green revolution’s role in Africa, low adoption rates of agricultural technologies persist in SSA, suggesting a trap in traditional methods (Matsumoto et al. 2013). This may suggest that Africa continues to be trapped in traditional farming systems and hence lags in agricultural technological advancements. A study in Northern Ghana by Tanko and Ismaila (2021) underscored the significance of cultural and religious practices in determining technical efficiency. They recommended integrating culture and religion into agriculture technology diffusion for enhanced technical efficiency. Therefore, all REFILS stakeholders need to recognise social and culturally sensitive factors in agricultural technology development and dissemination.

#### Limited access to ICTs in smallholder livestock farming systems

In Africa, constrained access to ICTs presents a substantial barrier to effective REFILS in smallholder livestock farming systems. According to Mapiye et al. (2021) and Abdulai et al. (2023), smallholder farmers have limited access to research-based innovations in rural farming areas due to their lack of internet connectivity. The obstacles extend to the presence of weak foundational elements, such as low literacy levels, a shortage of digital skills, and restricted access to digital resources (Abdulai et al. 2023). These impede the seamless flow of knowledge between research institutions, extension services, farmers, and other stakeholders, impeding agricultural development efforts. Also, despite the demonstrated potential for ICTs such as mobile and internet-based solutions to transform public extension services (FAO 2020; Mapiye et al. 2021), there is a notable lack of concerted efforts to harness this potential (Abdulai et al. 2023). Therefore, for a more effective pluralistic extension, policies should promote the development and use of ICTs to bridge collaboration gaps between REFILS actors and to ensure the swift flow of new research findings to smallholders.

### Strengthening REFILS in Africa’s livestock production industry

Addressing factors contributing to a weakened research-extension-farmer-input linkage is crucial for the development and sustainability of Africa’s livestock farming. Invariably, this is because strengthened REFILS can unlock the potential of the farming sector, improve farmers’ livelihoods, and contribute significantly to economic development and food security (Swanson and Rajalathi 2010; Raidimi and Kabilit 2017). According to FAO (2017) agricultural extension services are essential to poverty reduction and rural development. However, this requires complementary efforts from all stakeholders involved in the livestock production system (Sigman 2016). Strengthening this linkage is crucial because the challenges affecting smallholder livestock farmers are systemic and can be effectively addressed through collective action (World Bank 2017). Access to research-backed information and innovations enables farmers to make informed decisions, leading to improved productivity and sustainability. A combination of policy changes and strategies to ensure that research-based innovations effectively reach livestock farmers are discussed in this review, drawing on successful approaches from the continent and elsewhere.

#### Promoting public-private partnerships

Managing research, extension, and timely dissemination of research technology to smallholder farmers requires collaboration beyond the public sector. A pluralistic, multi-agency approach that engenders the involvement of the private sector, farmer organisations, NGOs, cooperatives, professionals, agribusinesses, self-help groups, input dealers, the media, and IT is vital (Swanson and Rajalathi 2010; Raidimi and Kabiti 2017; Nwafor et al. 2021). Each entity contributes based on its strengths and capabilities to create effective and sustainable synergies. A study by Zahran et al. (2020) brought new insights into the need for strengthening public-private partnerships to enhance AKIS functioning. Thus, the current focus has metamorphosed from a centralised, top-down, and supply-driven approach (Alex et al. 2002) to demand and market-oriented systems approach (Agwu et al. 2023). More importantly, promoting the involvement of private partners fosters an enabling environment for private sector investments in agricultural extension, leading to increased provision of client-responsive and market-oriented research and advisory services (Nwafor et al. 2021).

To keep all stakeholders in the loop and enhance agricultural practices, regular information-sharing meetings involving research, extension, and private sector representatives are recommended. For example, in India, initiatives like the Agricultural Technology Management Agency (ATMA) involve private sector players in reviewing and assessing extension work plans (Swanson and Rajalathi 2010). Also, the National Institute of Agricultural Extension Management (MANAGE) conducts one-year training courses for input suppliers to foster strong PPPs and strengthen their extension skills. In African countries, FAO has developed capacities to innovate and bring together various stakeholders in agricultural industries (FAO 2020). The organisation has been running a 5-year EU-funded project called “Developing Capacities in Agricultural Innovation Systems: Scaling Up the Tropical Agriculture Platform Framework” in Burkina Faso, the Democratic Republic, Malawi, Rwanda, and Senegal since 2019. Therefore, such well-funded and managed projects could be used to push the REFILS agenda. The Philippines’ PPPs for Agriculture program facilitates collaborations between the government, private sector, and civil society organisations to enhance agricultural productivity and extend technologies to farmers (Philippines Government 2012). Also, such PPP-based projects are entitled to fiscal incentives under the country’s 2011 Investment Priorities Plan (IPP). In the development of agricultural technology innovation systems, Hermans et al. (2019) and Zahran et al. (2020) suggested that PPPs are ideal as systemic policy tools. The reason is that they drive important innovation system functions like knowledge development, networking, and search guidance in the initial stages, leading to stronger REFILS.

#### Implementing evidence-based policy and regulatory reforms

One way of solving the challenge of weak REFILS effectively as highlighted by many studies (Cai and Davis 2017; MacNairn 2018; Agwu et al. 2023), is to reconsider and reshape policies and frameworks that establish meaningful connections among its stakeholders, each possessing complementary expertise. Strong policy structures and regulatory frameworks coupled with strong leadership create an enabling environment that supports effective coordination and collaboration (Saravanan and Suchiradipta 2017; Zwane 2020; Agwu et al. 2023). Hermans et al. (2019) noted that PPPs have become increasingly popular within the mix of systemic policy instruments aimed at promoting AIS. This would mean that countries with supportive policies towards PPPs will have stronger REFILS in Africa. Ethiopia has made progress due to its focus on policy reforms, and strong implementation mechanisms seeking to strengthen the relationship between government bodies, research institutions, and NGOs (Bealy and Dawit 2017; Davis et al. 2020) (Table 2). Also, India’s AIS approach and fodder innovation project is proving effective with its increased institutional pluralism allowing effective coordination and mutual benefits among all actors working with livestock farmers (Saravanan and Suchiradipta 2017). Apart from these, there is a need to explore policies on the applicability of the market systems approach to REFILS. Nwafor et al. (2021) demonstrated how the market systems approach, coupled with private sector involvement, capitalised on existing market opportunities to deliver necessary services alongside major agri-businesses.

On the other hand, regulatory reforms should be streamlined to facilitate the registration and commercialisation of new agricultural technologies and inputs. Evidence-based regulatory decisions ensure farmers have access to innovative solutions. India’s National Agricultural Policy focuses on evidence-based policymaking and the removal of unnecessary regulatory barriers to promote agricultural growth (Organization for Economic Cooperation and Development (OECD), 2022). It emphasises technology adoption, encourages private sector participation, and eliminates marketing barriers to support research and extension for farmers.

Addressing weak REFILS requires a strategic focus on policy and regulatory reforms. Studies suggest that reshaping policies to establish meaningful connections among stakeholders, coupled with strong leadership, creates an enabling environment for effective coordination and collaboration.

#### Strengthening the participation of livestock farmers and farmer organisations

Supporting the formation and capacity-building of farmer organisations and cooperatives and their active participation is key to strengthening REFILS (Singh et al. 2019). These entities can serve as intermediaries between research institutions, extension services, markets, and individual farmers, facilitating research-based and demand-driven solutions and practices (Agwu et al. 2023). According to Singh et al. (2019), utilising participatory technology generation, conducting monitoring and evaluation in research and extension activities, along with the establishment of village knowledge centres and farmers’ organisations can enhance REFILS and market connections for farmers. Participatory approaches, like FFSs, encourage farmers’ engagement and foster research-backed practices (Eicher 2007). EuroDairy, through its multi-actor EuroDairy approach, works on research-driven approaches to reduce antibiotic use in dairy farming. Such projects focusing on individual farmers and farmers’ groups continue to promote new knowledge exchange and learning among the farmers and between farmers and experts (Agwu et al. 2023). Despite limited improvement in research and public-sector extension coordination through Malawi’s ‘Pig sector innovation platforms’, government-level efforts and interests have strengthened linkages by professionalising cooperatives, promoting public extension, and establishing livestock farmer associations (Sigman 2016). Furthermore, the Industrial Development Corporation (IDC) Nguni cattle farmer programme in South Africa has successfully implemented an arrangement involving the government’s provincial departments of agriculture, local universities, the funding institution (IDC), and the groups of emerging smallholder livestock farmers (Mapiye et al. 2019). These entities worked collectively with the farmers’ groups to support research and technology development and information dissemination. Therefore, farmers’ active involvement in decision-making processes and participation in knowledge creation and sharing initiatives could significantly influence the success of REFILS.

#### Investing in ICTs

Going forward, the success of initiatives like REFILS hinges significantly on embracing the digital revolution. Many studies (World Bank 2017; FAO 2017; Abdulai et al. 2023), underscore the transformative potential of ICTs, including web-based and mobile applications, in enhancing the efficiency of information dissemination to smallholder farmers. Mapiye et al. (2019), reported a rise in demand for authentic and localised agricultural information from smallholder livestock farmers across Africa. Therefore, African governments and organisations must prioritise investments in digital agriculture, expanding mobile-based advisory services, and ensuring access to affordable smartphones and improved internet connectivity in rural areas (Mapiye et al. 2021).

The ongoing efforts in East Africa showcase the positive impact of ICT-based initiatives, and it is crucial to extend and replicate such successes across the entire continent (Tsan et al. 2019). The integration of ICT facilitates farmer-to-farmer interactions, as well as two-way communication between farmers and researchers (FAO 2017; Tsan et al. 2019). This promotes the development of farmer-driven solutions capable of addressing heterogeneous and complex farm management issues within the smallholder sector. According to Amadu et al. (2015), mobile applications like Community Knowledge Workers (CKWs) are making a significant impact, particularly in remote areas following the “last mile principle.” This approach facilitates the dissemination of agricultural knowledge to farmers in areas where poor road networks and inadequate infrastructure typically hinder government extension agents from reaching. As these ICT-based initiatives continue to evolve, emphasising user involvement in the development process becomes paramount for sustainable and impactful implementation in research and extension programmes (FAO 2020). Some of the applications being used to improve extension services delivery in SSA include M-PESA and Esoko (World Bank 2017). The global landscape also reflects notable initiatives, such as the USDA’s AgLearn system (USDA, 2021) as well as FAO’s Technologies and Practices for Small Agricultural Producers (TECA) and TAPipedia (FAO 2020), demonstrating the commitment to leveraging digital innovation to facilitate the rapid dissemination of research findings and best practices.

In essence, the strategic integration of ICTs into REFILS is indispensable for advancing collaborative research knowledge generation and ensuring the effective adoption of research-based technologies and best practices in the pursuit of agricultural sustainability within smallholder farming systems.

## Conclusions

The review paper critically examines REFILS in the context of African livestock farming, shedding light on the underlying concepts, challenges and opportunities for its improvement. It highlights the substantial influence of historical concepts and evolving methodologies in shaping knowledge creation and transfer theories that underpin REFILS. Also, the evolution of agricultural research and livestock extension from centralised top-down and supply-driven models to participatory and demand-driven approaches is critical in emphasising inclusivity, sustainability, innovation, and market orientation. Moreover, research agendas and practices should focus not only on livestock farmers and extension, but also on markets, policies, people, and environmental concerns. On the other hand, EAS continues to evolve but is still overwhelmed by challenges hindering its collaboration with other key REFILS actors like researchers, input producers, and farmers. This paper highlights the importance of adaptive and responsive EAS as catalysts that allow agricultural innovation to be tailored to the unique needs of smallholder livestock farming communities.

REFILS implementation faces hurdles such as inadequate funding, limited access to qualified personnel, poor policies, lack of ICT infrastructure, and essential inputs. These challenges create barriers that hinder the seamless collaboration between research institutions, extension services, input producers, and farmers. Addressing these challenges requires concerted efforts from all stakeholders and the need to adopt and adapt existing frameworks supporting REFILS to country and local needs and objectives. To achieve the intended goal of transforming REFILS, policy strategies and frameworks that link all stakeholders in a collaborative and sustainable manner should be recognised. This also includes the need for culturally sensitive approaches integrating local traditions and beliefs into agricultural technology development and transfer. Additionally, the strengthening REFILS through public-private partnerships, enhancing farmer participation, and markets are pivotal strategies. Moreover, the capacity of ICTs to bring refreshed momentum to REFILS appears even more compelling in view of the growing need for investments in agricultural research, strong interests from the private sector in the advancement of ICTs, and the call for improving the sustainability of smallholder livestock farming in Africa.

## Data Availability

The data generated and/or analysed during the current study are available upon request to the corresponding author.
